# Depression does not moderate the association between beliefs about medicines and medication adherence in patients with rheumatoid arthritis: A longitudinal study

**DOI:** 10.1177/13591053251345584

**Published:** 2025-06-25

**Authors:** Hermann Szymczak, Susanne Brandstetter, Boris Ehrenstein, Mark Steinmann, Christian Apfelbacher

**Affiliations:** 1 Otto von Guericke University Magdeburg, Germany; 2 University of Regensburg, Germany; 3 Asklepios Medical Center Bad Abbach, Germany; 4 DONAUISAR Klinikum Deggendorf, Germany

**Keywords:** beliefs about medicines, depression, longitudinal study, medication adherence, moderation analysis, rheumatoid arthritis

## Abstract

Non-adherence is common in people with rheumatoid arthritis (RA). According to the ‘necessity-concerns framework’, beliefs about the necessity and concerns about prescribed medications influence adherence. The present study’s objective was to investigate whether depression moderates associations between beliefs about medicines and adherence among people with physician-diagnosed RA in a longitudinal study (*N* = 361; 31% male, mean age [SD] 60.2 [13.4]). Adherence (adherent vs non-adherent; MARS), beliefs about medicines (BMQ), and depression (HADS) were assessed at baseline, 3- and 12-month follow-up. Multivariate logistic regression models were calculated (predictors: necessity/concerns, depression; outcome: adherence). Two-thirds of patients were non-adherent. No significant interactions were found between necessity/concerns and depression. Contrary to previous cross-sectional research, we did not find evidence for a moderating role of depression in associations between beliefs about medicines and adherence. Possible moderating effects of depression might be less robust than previously thought and therefore harder to detect in longitudinal analyses.

## Background

Rheumatoid arthritis (RA) is a chronic autoimmune disease characterized by an often progressive course. Patients with RA suffer from pain, joint swelling, limited mobility, associated disability and reduced health-related quality of life (Di Matteo et al., 2023; Smolen et al., 2018). Although pharmacological treatment is effective, non-adherence is prevalent in patients with RA. Estimates of the extent of non-adherence vary between 10% and 90% (Anghel et al., 2018; Scheiman-Elazary et al., 2016; van Den Bemt et al., 2012).

The ‘necessity-concerns framework’ (Horne and Weinman, 1999) explains non-adherence in terms of beliefs about medicines, that is, patients’ beliefs about (a) the *necessity* of their prescribed medications to maintain their health and (b) their *concerns* about potential side effects. This association between beliefs about ones’ prescribed medication and adherence is empirically well established: Several reviews on the causes of non-adherence in the treatment of RA have identified patients’ beliefs about the necessity of medication as well as patients’ concerns consistently as relevant patient-centred factors associated with non-adherence (Chowdhury et al., 2022; Horne et al., 2013; Pasma et al., 2013).

An important area of research is the investigation of the way in which the association between beliefs about medicines and adherence behaviour may be influenced by patient characteristics, such as the presence of co-morbidities. This way, interventions to improve adherence in RA patients can be better tailored towards patients’ needs.

A common co-morbidity in RA patients is depression (Cheng et al., 2023; Matcham et al., 2013). In a cross-sectional analysis, Brandstetter et al. (2016) found that depression moderates the relationship between beliefs about medicines and adherence in a sample of RA patients, with different effects depending on the specific beliefs in question (necessity beliefs vs concerns). Specifically, patients with elevated depressive symptoms showed a stronger association between necessity beliefs and adherence, whereas higher depressive symptoms attenuated the associations between concern and adherence. This study was limited by its cross-sectional study design. Cross-sectional findings may overestimate the actual effect size, for example, as a methodological artefact due to co-occurrence of predictor and outcome measures. Therefore, longitudinal studies of patient cohorts are needed to establish the robustness of results from cross-sectional analyses (and also strengthen causal assumptions). Our research question therefore is whether depression moderates the association between beliefs about medications and medication adherence in patients with RA in a longitudinal setting using the same cohort as Brandstetter et al. (2016).

## Methods

### Study design and sample

The study was conducted at the Department of Rheumatology, Asklepios Medical Center Bad Abbach, a tertiary care centre for patients with autoimmune and rheumatic diseases, located in the south of Germany (Brandstetter et al., 2016, 2017). At the initial study visit (baseline [T0]), participants were asked to complete self-report questionnaires. In addition, information was extracted from electronic health records. Patients were eligible for participation if they were aged 18 years or older, had physician-diagnosed RA and were currently under medical treatment for RA. Baseline data were collected in 2012 and 2013 (T0, *N* = 361). At baseline, participants provided their postal address and received self-report questionnaires by mail 3 (T1, *N* = 292) and 12 (T2, *N* = 245) months later as follow-ups. The study has been approved by the ethics committee of University of Regensburg (file-number: 12-101-0126).

### Measures

Medication adherence, beliefs about medicines, and depression were assessed at all three measurement points.

#### Medication adherence

Medication adherence was assessed using the German version of the Medication Adherence Report Scale (MARS; Mahler et al., 2010). The scale consists of five items on intentional and non-intentional adherence behaviours (e.g. forgetting to take medication, or intentionally taking less dosage than prescribed). Items are answered on a five-point Likert-scale ranging from 1 (never) to 5 (always). A sum score was calculated (Cronbach’s α = 0.769, 0.773 and 0.791 for baseline, 3 and 12 months follow-up). Due to the highly skewed distribution, we dichotomized the MARS score with the value of 25 indicating complete adherence (vs 24 or less indicating non-adherence). Additionally, sensitivity analyses were performed with cut-off values of ≤24 and ≤23 to ensure the robustness of the results (data not shown). As there is no agreement in the literature on a specific cut-off value, we applied sensitivity analyses to confirm the main results of the analyses by repeating the analyses with varying cut-off points (i.e. 23, 24 and 25). This approach also encapsulates a median split (Md = 24 [IQR = 22–25]) as a statistical method for dichotomization for all MARS scores at baseline, and 3 and 12 months follow-up.

#### Beliefs about medicines

Patients’ beliefs about medicines were measured using two scales of the Beliefs about Medicines Questionnaire (BMQ; Horne et al., 1999; Mahler et al., 2012). The ‘necessity’ scale assesses patients’ perceived necessity regarding their prescribed medicines and consists of five items (e.g. ‘I could not live without my medicines’). The scale ‘concern’ assesses patients’ concerns about their medicines and consists of six items (e.g. ‘Sometimes, I worry about my medicines’). Items are answered on a five point Likert-Scale from 1 (fully disagree) to 5 (fully agree). The scale scores were calculated as a mean with higher values indicating stronger beliefs. Cronbach’s alpha were α = 0.766, 0.858 and 0.856 (necessity) and α = 0.758, 0.770 and 0.798 (concerns), for baseline, 3 and 12 months, respectively.

#### Depression

The depression scale of the Hospital Anxiety and Depression Scale (HADS; Zigmond and Snaith, 1983) was used to assess patients’ depressiveness. The total sum score ranges from 0 to 21, with higher values indicating the presence of more depressive symptoms (Cronbach’s α = 0.861, 0.886 and 0.870, for baseline, 3 and 12 months, respectively).

### Statistics

For continuous variables, either median (Md) or interquartile range (IQR), or mean (*M*) and standard deviation (SD) are reported. For categorical variables, counts and percentages are used. Group differences were determined by *t*-tests.

Multivariate logistic regression models were calculated to test the moderating role of depression. To ensure comparability with Brandstetter et al. (2016), analyses were replicated from that study: Necessity/concerns and depression are predictor variables, and were transformed by grand mean centring before inclusion into the regression model. Sex (for necessity only), the number of prescribed RA medications and self-rated pain were included as confounders; in the original study, the included confounders were selected from a larger set of theoretically devised confounders, based on univariate analyses. Results of the logistic regression analyses are given as odds ratios (OR) and 95% confidence intervals (CI). Model fit is reported as Nagelkerke’s *R*^2^. *p*-Values <0.05 were interpreted as statistically significant. All analyses were performed using SPSS.29. To assess the robustness of the association between the multivariable predictors and non-adherence, the performance of the non-adherence model was tested with a less conservative definition (MARS values ≤ 24 and ≤23; data not shown).

For the regression models, beliefs about medicines (necessity, concerns) at T0 predict adherence at T1 and T2. The stability of beliefs about medicines is then assessed (T0–T1). If beliefs about medicines are not sufficiently stable (i.e. low reliability), regression models are calculated with beliefs about medicines at T1 predicting adherence at T2. If reliability is high (T0–T1: *r* ≥ 0.7), this step is omitted.

## Results

### Sample characteristics

Characteristics of study participants are presented in Table 1. Variables include socio-demographic and medical characteristics. About 30% of participants were male with a mean age of approximately 60 years (SD = 13.4, range: 19–92 years). The reported duration of RA was *M* = 11 years (SD = 9.2, range: <1–43 years), and about 40% of participants reported having experienced severe side effects from their medication at some point in time (‘Have you ever experienced serious side effects from the medication you take to treat your rheumatic disease?’).

**Table 1.** Sociodemographic and medical characteristics of study participants at Baseline (T0, *N* = 361).

Variable	*N*	Result
Sex male, *N* (%)	361	110 (30.5)
Age (years) (*M*, SD)	359	60.2 (13.36)
Migration background (%)	361	7.2
Schooling ≥ 10 years (%)	361	39.4
BMI (*M*, SD)	357	27.75 (5.80)
Age (years) at diagnosis (*M*, SD)	331	49.26 (15.21)
Duration of disease	330	10.94 (9.22)
Inpatient treatment (%)	360	21.7
Distance to provider of RA treatment (km) (*M*, SD)	357	58.59 (44.75)
Experience of severe side effects (% yes)	361	43.2
Number of prescribed medicines (*M*, SD)	360	7.13 (3.68)
Number of prescribed RA medicines (*M*, SD)	360	3.86 (1.60)
NSAR (any, %)	360	16.4
Opioids (any, %)	360	14.4
Corticosteroids (any, %)	360	71.9
DMARDs (any, %)	360	75.8
Biologicals (any, %)	360	29.2
Self-rated pain (1–10; *M*, SD)	359	4.47 (2.78)
DAS28 (*M*, SD)	361	3.07 (1.57)

M: mean; SD: standard deviation; RA: rheumatoid arthritis; BMI: body mass index; NSAR: non-steroidal anti-inflammatory drug; DMARDs: disease-modifying antirheumatic drugs; DAS28-joint Disease Activity Score (disease severity).

### Main outcomes

Descriptive results for BMQ, HADS Depression and MARS are presented in Table 2. Results are displayed for the complete sample as well as differentiated by adherence (adherent vs non-adherent). At all measurement points, 66%–68% of patients were non-adherent. Necessity beliefs, concerns and depression scores are also stable for the complete sample across measurement points (necessity: *M* = 4.2–4.3 [SD = 0.6–0.8]); concerns: *M* = 2.7–2.9 [SD = 0.8]; depression: *M* = 6.0–6.2 [SD = 3.8–4.0]). No group differences for necessity beliefs were found (all *p*s > 0.05). For concerns and depression, scores were significantly higher in non-adherent participants (Concerns: T0: *t*(341) = −3.71, *p* < 0.001, Cohen’s *d* = 0.43; T1: *t*(274) = −3.03, *p* < 0.01, Cohen’s *d* = 39; T2: *t*(236) = −2.58, *p* < 0.05, Cohen’s *d* = 0.36); Depression: T0: *t*(343) = −3.25, Cohen’s *d* = 0.38, *p* < 0.001; T1: *t*(279) = −3.65, *p* < 0.01, Cohen’s *d* = 0.47; T2: *t*(237) = −2.24, *p* < 0.05, Cohen’s *d* = 0.31).

**Table 2.** Descriptive results of main variables: Beliefs about medicines, depression and adherence.

Variables	Baseline (T0)			3 months (T1)			12 months (T2)		
	All	Adherent	Non-adh.	All	Adherent	Non-adh.	All	Adherent	Non-adh.
*N*	361	112	239	292	91	193	245	81	159
BMQ necessity (*M*, SD)	4.27 (0.64)	4.37 (0.58)	4.23 (0.65)	4.18 (0.73)	4.30 (0.64)	4.12 (0.77)	4.12 (0.79)	4.21 (0.81)	4.08 (0.78)
Valid *(N)*	355	111	238	285	89	191	241	81	158
BMQ concerns (*M*, SD)	2.84 (0.80)	2.60 (0.84)	2.94 (0.75)*	2.90 (0.77)	2.70 (0.82)	3.0 (0.74)*	2.84 (0.83)	2.64 (0.85)	2.93 (0.80)*
Valid *(N)*	348	109	234	280	88	188	239	79	159
HADS depression (*M*, SD)	6.03 (3.83)	5.01 (3.99)	6.41 (3.62)*	6.20 (3.96)	4.97 (3.85)	6.76 (3.87)*	6.09 (3.89)	5.25 (4.13)	6.43 (3.73)*
Valid *(N)*	354	110	235	287	91	190	243	80	159
MARS (% non-adherence)	68			68			66		
Valid *(N)*	351			284			240		

*M*: mean; SD: standard deviation; BMQ: beliefs about medicines (necessity and concerns scale, range 0–5), HADS: Hospital Anxiety and Depression Scale (range 0–20); MARS: Medication Adherence Report Scale (dichotomized: adherence vs non-adherence).

* *t*-test (adherent–non-adherent): *p* < 0.05.

### Moderation analyses

Table 3 shows the results for the moderation analyses (multivariate logistic regressions). Main effects were significant only for depression (HADS) at T1 (ORs = 0.9, *p*s ≤ 0.02), whereas no other main effects or interactions (depression × necessity/concerns) were significant (all *p*s > 0.15).

**Table 3.** Moderation analysis of medication adherence on depression and necessity/concerns.

Variables	MARS (T1)			MARS (T2)		
	OR	95% CI	*p*	OR	95% CI	*p*
Necessity (T0)						
HADS depression	0.88	0.81, 0.96	**0.002**	0.92	0.85, 1.01	0.064
BMQ necessity	1.21	0.74, 1.98	0.440	1.19	0.74, 1.92	0.478
IA: depression × necessity	1.04	0.92, 1.18	0.558	0.95	0.86, 1.06	0.387
*N*			276			232
Nagelkerke’s *R*^2^			0.08			0.04
Concerns (T0)						
HADS depression	0.90	0.83, 0.98	**0.013**	0.96	0.87, 1.05	0.316
BMQ concerns	0.80	0.54, 1.18	0.265	0.75	0.50, 1.12	0.156
IA: depression × concerns	0.95	0.86, 1.05	0.282	0.99	0.89, 1.09	0.771
*N*			273			229
Nagelkerke’s *R*^2^			0.08			0.05

Logistic regression analysis of medication adherence (Medication Adherence Report Scale (MARS); predictors were mean centred; adjusted for sex (only necessity), number of prescribed rheumatoid arthritis (RA) medicines (T0), self-rated pain (T0). Bold printed *p*-values significant (*p* < −05).

IA: interaction; OR: odds ratio; CI: confidence interval; HADS: Hospital Anxiety and Depression Scale; BMQ: Beliefs about Medicines Questionnaire.

Reliability of BMQ measures and HADS depression from T0 to T1 is high (necessity: *r* = 0.758, *p* < 0.001; concern: *r* = 0.694, *p* < 0.001, HADS depression: *r* = 0.798, *r* < 0.001) and therefore no additional moderation analyses (BMQ, HADS depression T1 to MARS T2) were computed. Furthermore, the interaction term remains non-significant in all sensitivity analyses (all *p*s > 0.16).

## Discussion

### Key findings

We did not find a moderating role of depression for the association between beliefs about medicine and adherence behaviour in a sample of people with RA. Thus, the key finding of the cross-sectional moderation analysis conducted by Brandstetter et al. (2016) could not be replicated in a longitudinal approach utilizing the same cohort study sample.

### Strengths and limitations

Main strengths of the present study include a well characterized cohort with little missing data and the prospective design, with a relatively low dropout rate (68% of patients at 1-year follow-up) and a corresponding longitudinal analyses of the relationship between beliefs and behaviour across measurement points. This approach is essential for validating findings from cross-sectional analyses and investigate causal assumptions. A further strength of the present study is the relatively representative socioeconomic (i.e. educational) status of the sample: 60% of sample reported to have 10 or less years of schooling, compared to 66% of the German general population (Bundeszentrale für politische Bildung, 2024). A representative sample with regard to SES is especially important since SES, or education respectively and medication use in RA patients are associated in (e.g. Russell et al., 2023).

One of the main limitations of the study is the use of self-report to assess adherence. Self-reporting of medication adherence can be biased, for instance due to recall bias or social desirability, which could lead to an overestimation of true adherence (Stirratt et al., 2015). In addition, the dichotomization of our adherence measure could result in more subtle influences remaining undetected (Pasma et al., 2013).

### Interpretation, in relation to literature

The study by Brandstetter et al. (2016) was the first to examine the potential role of depression as a moderator within the ‘necessity-concerns’ framework. We followed up on their study, but with different results. Our non-significant findings may indicate that the moderating effect of depression may be more elusive than initially expected. To the best of our knowledge, no other studies have yet investigated this research question in patients with RA.

Conversely, there is a body of literature examining the direct influence of depression on non-adherence in RA patients. This literature predominantly indicates an association between depression and non-adherence (Chowdhury et al., 2022; Goh et al., 2017; Vangeli et al., 2015) For instance, in their systematic review of factors associated with non-adherence to treatment for immune-mediated inflammatory diseases, Vangeli et al. (2015) conclude that treatment concerns, necessity beliefs and depression are among the psychological factors with the strongest evidence for an impact on adherence. In this regard, it is noteworthy, that the only significant effects identified in our study were observed for depression predicting adherence at the 3-month follow-up. Consequently, depression may be modelled as a direct influence on adherence rather than a moderator (Chowdhury et al., 2022; Dagli et al., 2024; Vangeli et al., 2015). Moreover, recent studies suggest a bidirectional relationship between depression and rheumatoid arthritis (Lwin et al., 2020; Ng et al., 2022) highlighting a dynamic interplay between adherence and depression that encompasses both biological and psychological factors.

One potential explanation for the lack of significance in our findings is that empirical effect sizes are reduced to the longitudinal design, whereby the predictor and outcome variables are not measured at the same time. For instance, the ORs for the interaction effects are slightly lower in comparison to those reported by Brandstetter et al. (2016): ORs: 1.08–1.15 versus 0.95–1.04. In addition to this methodological issues, publication bias may also be a contributing factor in explaining the discrepancy between our findings and those of the majority of existing studies with regard to the main effects of necessity/concerns.

Furthermore, it is possible that the results are influenced by selective dropout. For example, patients with lower levels of adherence or higher scores on depression may be more likely to drop out. However, dropout analyses showed that there were no statistically significant differences between study sample and drop-out sample with regard to beliefs (necessity, concerns) and non-adherence at baseline (*t*-tests and chi-squared tests, all *p*s > 0.05). Also, no baseline difference was identified for the 3-month dropout sample in terms of depression (*p* > 0.05). However, at the 12-month mark, the dropout sample exhibited slightly elevated depression scores at baseline in comparison to the study sample (HADS depression = 6.8 vs 5.7, *t* = 2.4, *p* = 0.019). We then repeated the interaction analyses of Brandstetter et al. (2016) with only the subset of participants who remained at the 12-month follow-up (*N* = 245). In this analysis, the interaction term did not reach statistical significance (OR = 1.17, *p* = 0.057). This suggests that selective dropout of patients with higher depressive symptoms could contribute to the non-significant findings we observed in the long term.

### Future research

Further prospective cohort studies are needed. These studies should ideally use more direct measures of adherence, in conjunction with self-report data. For example, measurement of blood drug levels or measurement methods based on regular, daily or event-based monitoring could be utilized, such as diaries or medication event monitoring systems (MEMS; Michaud et al., 2019). Other psychological comorbidities, such as anxiety, could also be investigated as potential moderators of the relationship between beliefs and adherence behaviour. Furthermore, the association between disease activity and adherence might also play a role in explaining adherence, as adherence impacts disease activity, and possibly vice versa (Hope et al., 2016; Kelly et al., 2020; Li et al., 2017).

As RA is a chronic disease, a cohort study of patients should be conducted over several years, ideally from the start of therapy. In this way, influences of factors such as comorbidities, treatment outcomes (e.g. remission), discontinuation or change of therapy, etc. can be studied in their interaction with changes in beliefs, and corresponding changes in behaviour can be evaluated together in their ‘true’ (long-term) dynamics. In this way, the actual impact of various potentially influential factors (such as depression) can be identified in a more realistic setting.

### Conclusions

Contrary to previous research, we did not find evidence for a moderating role of depression in the association between beliefs about necessity or patients’ concerns and adherence behaviour in a longitudinal setting. Thus, possible moderating effects of depression might be less robust than previously thought and therefore harder to detect in longitudinal analyses. Further longitudinal studies are warranted to evaluate the robustness of cross-sectional findings, including different populations and settings.

## References

[bibr1-13591053251345584] Anghel L-A Farcaş AM Oprean RN (2018) Medication adherence and persistence in patients with autoimmune rheumatic diseases: A narrative review. Patient Preference and Adherence 12: 1151–1166. 30013327 10.2147/PPA.S165101 PMC6037147

[bibr2-13591053251345584] Brandstetter S Riedelbeck G Steinmann M , et al. (2016) Depression moderates the associations between beliefs about medicines and medication adherence in patients with rheumatoid arthritis: Cross-sectional study. Journal of Health Psychology 23(9): 1185–1195. 27151068 10.1177/1359105316646440

[bibr3-13591053251345584] Brandstetter S Riedelbeck G Steinmann M , et al. (2017) Pain, social support and depressive symptoms in patients with rheumatoid arthritis: Testing the stress-buffering hypothesis. Rheumatology International 37(6): 931–936. 28124095 10.1007/s00296-017-3651-3

[bibr4-13591053251345584] Bundeszentrale für politische Bildung (2024) Zahlen und Fakten: Die soziale Situation in Deutschland [Numbers and facts: The social situation in Germany]. Available at: https://www.bpb.de/kurz-knapp/zahlen-und-fakten/soziale-situation-in-deutschland/ (accessed 26 July 2024).

[bibr5-13591053251345584] Cheng L Gao W Xu Y , et al. (2023) Anxiety and depression in rheumatoid arthritis patients: Prevalence, risk factors and consistency between the hospital anxiety and depression scale and Zung’s self-rating Anxiety Scale/Depression Scale. Rheumatology advances in practice 7(3): rkad100. 10.1093/rap/rkad100 PMC10681850 38033365

[bibr6-13591053251345584] Chowdhury T Dutta J Noel P , et al. (2022) An overview on causes of nonadherence in the treatment of rheumatoid arthritis: Its effect on mortality and ways to improve adherence. Cureus 14(4): e24520. 10.7759/cureus.24520 PMC9136714 35651472

[bibr7-13591053251345584] Dagli A Lee R Bluett J (2024) The effect of depression on disease activity and teatment response in patients with inflammatory arthritis: Results from a narrative literature review. Neuropsychiatric Disease and Treatment 20: 1377–1386. 38988973 10.2147/NDT.S456231 PMC11233831

[bibr8-13591053251345584] Di Matteo A Bathon JM Emery P (2023) Rheumatoid arthritis. Lancet 402(10416): 2019–2033. 38240831 10.1016/S0140-6736(23)01525-8

[bibr9-13591053251345584] Goh H Kwan YH Seah Y , et al. (2017) A systematic review of the barriers affecting medication adherence in patients with rheumatic diseases. Rheumatology International 37(10): 1619–1628. 28681249 10.1007/s00296-017-3763-9

[bibr10-13591053251345584] Hope HF Bluett J Barton A , et al. (2016) Psychological factors predict adherence to methotrexate in rheumatoid arthritis: Findings from a systematic review of rates, predictors and associations with patient-reported and clinical outcomes. RMD Open 2(1): 1–15. 10.1136/rmdopen-2015-000171 PMC4731843 26848403

[bibr11-13591053251345584] Horne R Chapman SC Parham R , et al. (2013) Understanding patients’ adherence-related beliefs about medicines prescribed for long-term conditions: A meta-analytic review of the necessity-concerns framework. PLoS One 8(12): e80633. 10.1371/journal.pone.0080633 PMC3846635 24312488

[bibr12-13591053251345584] Horne R Weinman J (1999) Patients’ beliefs about prescribed medicines and their role in adherence to treatment in chronic physical illness. Journal of Psychosomatic Research 47(6): 555–567. 10661603 10.1016/s0022-3999(99)00057-4

[bibr13-13591053251345584] Horne R Weinman J Hankins M (1999) The beliefs about medicines questionnaire: The development and evaluation of a new method for assessing the cognitive representation of medication. Psychology and Health 14(1): 1–24.

[bibr14-13591053251345584] Kelly A Crimston-Smith L Tong A , et al. (2020) Scope of outcomes in trials and observational studies of interventions targeting medication adherence in rheumatic conditions: A systematic review. Journal of Rheumatology 47(10): 1565–1574. 31839595 10.3899/jrheum.190726

[bibr15-13591053251345584] Li L Cui Y Yin R , et al. (2017) Medication adherence has an impact on disease activity in rheumatoid arthritis: A systematic review and meta-analysis. Patient Preference and Adherence 11: 1343–1356. 28831245 10.2147/PPA.S140457 PMC5553350

[bibr16-13591053251345584] Lwin MN Serhal L Holroyd C , et al. (2020) Rheumatoid arthritis: The impact of mental health on disease: A narrative review. Rheumatology and Therapy 7(3): 457–471. 32535834 10.1007/s40744-020-00217-4 PMC7410879

[bibr17-13591053251345584] Mahler C Hermann K Horne R , et al. (2010) Assessing reported adherence to pharmacological treatment recommendations: Translation and evaluation of the medication adherence report scale (MARS) in Germany. Journal of Evaluation in Clinical Practice 16(3): 574–579. 20210821 10.1111/j.1365-2753.2009.01169.x

[bibr18-13591053251345584] Mahler C Hermann K Horne R , et al. (2012) Patients’ beliefs about medicines in a primary care setting in Germany. Journal of Evaluation in Clinical Practice 18(2): 409–413. 21087373 10.1111/j.1365-2753.2010.01589.x

[bibr19-13591053251345584] Matcham F Rayner L Steer S , et al. (2013) The prevalence of depression in rheumatoid arthritis: A systematic review and meta-analysis. Rheumatology 52(12): 2136–2148. 24003249 10.1093/rheumatology/ket169 PMC3828510

[bibr20-13591053251345584] Michaud K Vrijens B Tousset E , et al. (2019) Real-world adherence to oral methotrexate measured eelectronically in patients with established rheumatoid arthritis. ACR Open Rheumatology 1(9): 560–570. 31777840 10.1002/acr2.11079 PMC6858035

[bibr21-13591053251345584] Ng CYH Tay SH McIntyre RS , et al. (2022) Elucidating a bidirectional association between rheumatoid arthritis and depression: A systematic review and meta-analysis. Journal of Affective Disorders 311: 407–415. 35642835 10.1016/j.jad.2022.05.108

[bibr22-13591053251345584] Pasma A Van’t Spijker A Hazes JM , et al. (2013) Factors associated with adherence to pharmaceutical treatment for rheumatoid arthritis patients: A systematic review. Seminars in Arthritis and Rheumatism 43(1): 18–28. 23352247 10.1016/j.semarthrit.2012.12.001

[bibr23-13591053251345584] Russell O Lester S Black RJ , et al. (2023) Socioeconomic status and medication use in rheumatoid arthritis: A scoping review. Arthritis Care & Research 75(1): 92–100. 36106932 10.1002/acr.25024 PMC10100498

[bibr24-13591053251345584] Scheiman-Elazary A Duan L Shourt C , et al. (2016) The rate of adherence to antiarthritis medications and associated factors among patients with rheumatoid arthritis: A systematic literature review and metaanalysis. Journal of Rheumatology 43(3): 512–523. 26879354 10.3899/jrheum.141371

[bibr25-13591053251345584] Smolen JS Aletaha D Barton A , et al. (2018) Rheumatoid arthritis. Nature Reviews Disease Primers 4(1): 18001. 10.1038/nrdp.2018.1 29417936

[bibr26-13591053251345584] Stirratt MJ Dunbar-Jacob J Crane HM , et al. (2015) Self-report measures of medication adherence behavior: Recommendations on optimal use. Translational Behavioral Medicine 5(4): 470–482. 26622919 10.1007/s13142-015-0315-2 PMC4656225

[bibr27-13591053251345584] van Den Bemt BJ Zwikker HE van Den Ende CH (2012) Medication adherence in patients with rheumatoid arthritis: A critical appraisal of the existing literature. Expert Review of Clinical Immunology 8(4): 337–351. 22607180 10.1586/eci.12.23

[bibr28-13591053251345584] Vangeli E Bakhshi S Baker A , et al. (2015) A systematic review of factors associated with non-adherence to treatment for immune-mediated inflammatory diseases. Advances in Therapy 32(11): 983–1028. 26547912 10.1007/s12325-015-0256-7 PMC4662720

[bibr29-13591053251345584] Zigmond AS Snaith RP (1983) The hospital anxiety and depression scale. Acta Psychiatrica Scandinavica 67(6): 361–370. 6880820 10.1111/j.1600-0447.1983.tb09716.x

